# Non-Union of Bone during Pregnancy

**Published:** 1888-06

**Authors:** 


### Non-Union of Bone During Pregnancy.

M. Padieu, of Amiens, describes the case of a woman who fractured the tibia and fibula of one leg on January 4, 1886. The fracture was put up in a many-tailed bandage. At the end of a month it was found that there was still movement between the fragments, and a fortnight later there was no further progress. At the end of three months and a half, M. Padieu put the patient on crutches in order to hasten consolidation. Union was still delayed; then he began to suspect pregnancy. The patient admitted that her period had not recurred since December 26th, nine days before the accident. The abdomen and pelvis were examined, and M. Padieu's suspicions were confirmed. Delivery occurred on September 28th, and a well-nourished live child was born. Ten days after delivery the patient noticed that her leg felt much stronger than it had ever been since the accident. At the end of a month union was perfect. The *Archives de Tocologie* observes that this case was remarkably clear and authentic. The physiologist and obstetrician may advantageously consider the facts of the case. The delay in ossification of the callus must surely be due to some cause more subtle than mere debility. Indeed, the amount of debility involved in an average pregnancy is often very trifling. Severe abdominal operations, even double ovariotomy, have been tolerated with impunity during gestation, the wounds healing and delivery occurring at term. The theory that lime salts are used up during pregnancy for the benefit of the fœtus, and to the prejudice of the callus, will hardly bear strict examination, or at least it demands strong proof that local nutrition in the parent is palpably affected.—*Maryland Medical Journal.*

